# C-reactive protein-to-lymphocyte ratio and perforation in pediatric appendicitis

**DOI:** 10.1007/s00595-026-03345-6

**Published:** 2026-05-26

**Authors:** Zlatan Zvizdic, Asmir Jonuzi, Nadja Zvizdic, Semir Vranic

**Affiliations:** 1 Department of Pediatric Surgery, Clinical Center, University of Sarajevo, Sarajevo, Bosnia and Herzegovina; 2 Department of Anesthesiology and Intensive Care, Garmisch-Partenkirchen Medical Center, Garmisch-Partenkirchen, Germany; 3 College of Medicine, QU Health, Qatar University, PO Box 2713, Doha, Qatar

**Keywords:** Pediatric acute appendicitis, Appendiceal perforation, C-reactive protein–to–lymphocyte ratio, Inflammatory biomarkers, Risk stratification

## Abstract

**Purpose:**

Early distinction between perforated and non-perforated acute appendicitis (AA) in children remains challenging. Conventional inflammatory markers have limited accuracy, highlighting the potential value of composite inflammatory indices.

**Methods:**

Pediatric patients were categorized as having non-perforated or perforated appendicitis based on intraoperative findings and histopathologic confirmation. Laboratory data obtained on hospital admission included C-reactive protein (CRP), white blood cell count, and differential leukocyte percentages.

**Results:**

The subjects of this retrospective study were 338 pediatric patients, 48 (14.2%) of whom had perforated AA. The CRP to lymphocyte ratio (CRP/LR) was significantly higher in the patients with perforated AA (*p* < 0.001). ROC analysis demonstrated good discriminative performance of CRP/LR for predicting appendiceal perforation, with an area under the curve of 0.84 (95% CI 0.78–0.90). A CRP/LR cut-off value of ≥ 7.2 yielded a sensitivity of 83.3% and a specificity of 78.6%. After adjustment for age, symptom duration, and body temperature on admission, CRP/LR remained an independent predictor of appendiceal perforation in the multivariate regression analysis.

**Conclusion:**

The CRP/LR showed good diagnostic performance in differentiating perforated from non-perforated AA and may serve as a simple, readily available tool for early risk stratification in pediatric patients.

## Introduction

Acute appendicitis (AA) is one of the most common surgical emergencies in children, and diagnostic delays remain a challenge, often leading to significant morbidity. Children are more susceptible to disease progression because of the atypical clinical presentations that mimic other gastrointestinal illnesses, their limited ability to report symptoms clearly, and rapid development of the inflammatory response [1]. Consequently, appendiceal perforation occurs more frequently in pediatric patients than in adults, with reported rates ranging from 20% to over 40% and younger age consistently identified as a key risk factor for complicated disease [2, 3]. Perforation is associated with substantially worse outcomes, including intra-abdominal abscess, prolonged hospitalization, increased postoperative complications, and greater healthcare utilization.

Early identification of children at increased risk of appendiceal perforation is crucial for timely surgical intervention and optimal perioperative management, particularly because perforated AA represents an advanced and clinically complex stage of the disease, in which factors such as delayed presentation and intensified systemic inflammatory response contribute to severity and postoperative outcomes [4, 5]. In addition to these patient-related determinants, system-level factors, including access to care and the timing of surgical treatment, may also influence the risk of perforation and recovery after appendectomy, underscoring the importance of prompt diagnosis and intervention [6]. Although imaging techniques such as ultrasonography and computed tomography have improved diagnostic accuracy, their use in acute pediatric settings may be limited by operator dependence, radiation exposure, cost, and availability. Thus, laboratory inflammatory markers remain central to the initial evaluation of suspected AA, providing rapidly accessible information to support early risk stratification and clinical decision-making when imaging is inconclusive or delayed [7, 8].

Conventional inflammatory markers, such as white blood cell count and C-reactive protein (CRP), are used widely in clinical practice; however, when considered individually, their ability to reliably distinguish uncomplicated from perforated AA is limited [9, 10]. Although these markers indicate systemic inflammation, they lack the specificity needed to reflect disease severity accurately, underscoring the value of composite indices that capture the underlying inflammatory and immune responses in complicated AA.

There is growing interest in composite inflammatory indices derived from routine hematological parameters. Leukocyte-based ratios, particularly the neutrophil-to-lymphocyte ratio, have shown potential in predicting disease severity and appendiceal perforation in children [11, 12]. Lymphopenia reflects stress-related immune dysregulation, while elevated CRP indicates the extent of the systemic inflammatory response. Therefore, combining these complementary markers into a single index may offer a more clinically meaningful assessment of disease severity. The C-reactive protein–to–lymphocyte ratio (CRP/LR), also known as the C-reactive protein–lymphocyte ratio (CLR), is an emerging marker that integrates inflammatory burden with immune status. Although CRP/LR has demonstrated diagnostic and prognostic value in various inflammatory conditions, evidence supporting its use in pediatric AA, particularly for distinguishing non-perforated from perforated disease, remains limited [13]. Accordingly, this study evaluates the diagnostic performance of CRP/LR in differentiating non-perforated from perforated AA and compares its accuracy with that of conventional inflammatory markers. By addressing this gap, we sought to assess its potential role in early risk stratification in routine clinical practice.

## Materials and methods

This study was performed at a tertiary-care pediatric surgery center and included patients treated between January 2019 and December 2024. Children aged 2–18 years who underwent appendectomy for suspected AA were eligible for inclusion. Patients were divided into two groups based on intraoperative findings and histopathological examination: a non-perforated AA group and a perforated AA group. Perforation was defined by the presence of a visible appendiceal wall defect, localized or generalized peritonitis, periappendiceal abscess, or free purulent fluid in the abdominal cavity. Exclusion criteria included chronic inflammatory or autoimmune diseases, hematologic disorders, primary or secondary immunodeficiency, recent corticosteroid or immunosuppressive therapy, preoperative antibiotic treatment > 24 h, concurrent infections, malignancy, incomplete medical records, or missing laboratory data.

Demographic data (age and sex) and clinical information were extracted from the electronic medical records. Symptom duration was defined as the interval, in hours, from the onset of abdominal pain or other relevant symptoms to hospital admission. Body temperature on admission was measured using a standard axillary thermometer. Laboratory parameters obtained on admission before surgery included serum CRP (mg/L), total white blood cell (WBC) count, and differential leukocyte percentages (neutrophils and lymphocytes). All measurements were part of the routine clinical care. The neutrophil-to-lymphocyte ratio (NLR) was calculated by dividing the absolute neutrophil count by the absolute lymphocyte count, reflecting the balance between innate (neutrophil-mediated) and adaptive (lymphocyte-mediated) immune responses as a marker of systemic inflammation. The CRP/LR was calculated by dividing the serum CRP value (mg/L) by the lymphocyte percentage. This composite index integrates an acute-phase reactant with a measure of relative lymphocyte suppression, capturing complementary information on both systemic inflammatory burden and immune dysregulation. The primary outcome was the ability of the CRP/LR to distinguish between non-perforated and perforated AA. Secondary outcomes included comparisons of the CRP, lymphocyte count, total WBC, neutrophil percentage, and NLR between the two groups.

The study protocol was conducted in accordance with the Declaration of Helsinki and the Good Clinical Practice (GCP) guidelines [14]. All medical records were de-identified and anonymized for this study, which was approved by the local institutional review board (IRB) (Ethical Committee of the Clinical Center, University of Sarajevo, Approval number: 51-30-5-13298/23). The IRB also waived the requirement for informed consent because of the retrospective nature of the study.

### Statistical analysis

Continuous variables were assessed for normality using the Shapiro–Wilk test and presented as means ± standard deviation or as median (interquartile range) values, as appropriate. Between-group comparisons were performed using the Student’s t-test or the Mann–Whitney U test for continuous variables, and the chi-square or Fisher’s exact test for categorical variables. Receiver operating characteristic (ROC) curve analysis was used to evaluate the diagnostic performance of CRP/LR in predicting appendiceal perforation. The area under the curve (AUC) was calculated and the optimal cut-off value was calculated using the Youden index [15]. Sensitivity, specificity, positive predictive value, and negative predictive value were reported. Multivariate logistic regression analysis was performed to identify independent predictors of appendiceal perforation, adjusting for potential confounders, including age, symptom duration, and body temperature at admission. A two-tailed p-value < 0.05 was considered significant. All analyses were conducted using SPSS version 23.0 (IBM Corp., Armonk, NY, USA) and R Statistical Software version 4.3.2 (R Foundation for Statistical Computing, Vienna, Austria).

## Results

The study included 338 pediatric patients who underwent appendectomy for suspected AA. Based on intraoperative and histopathologic findings, 48 (14.2%) patients had perforated AA and 290 (85.8%) had non-perforated AA,. The groups were similar in age and sex distribution (*p* > 0.05 for both, Table 1).

**Table 1:** Baseline demographic and clinical characteristics of the cohort

Variable	Non-perforated appendicitis (*n* = 290)	Perforated appendicitis (*n* = 48)	*p*-value
Age, years, median (IQR)	11.8 (9.2-14.6)	12.1 (9.5-14.9)	0.64
Male sex, n (%)	182 (62.8)	31 (64.46)	0.81
Symptom duration ≥24 h, n (%)	104 (35.9)	29 (60.4)	<0.01

Data are presented as median values (interquartile range, IQR) or numbers (percentages), as appropriate. Comparisons between groups were performed using the Mann–Whitney U test for continuous variables and the chi-square test or Fisher’s exact test for categorical variables. A two-tailed p-value < 0.05 was considered significant

**Fig. 1:**
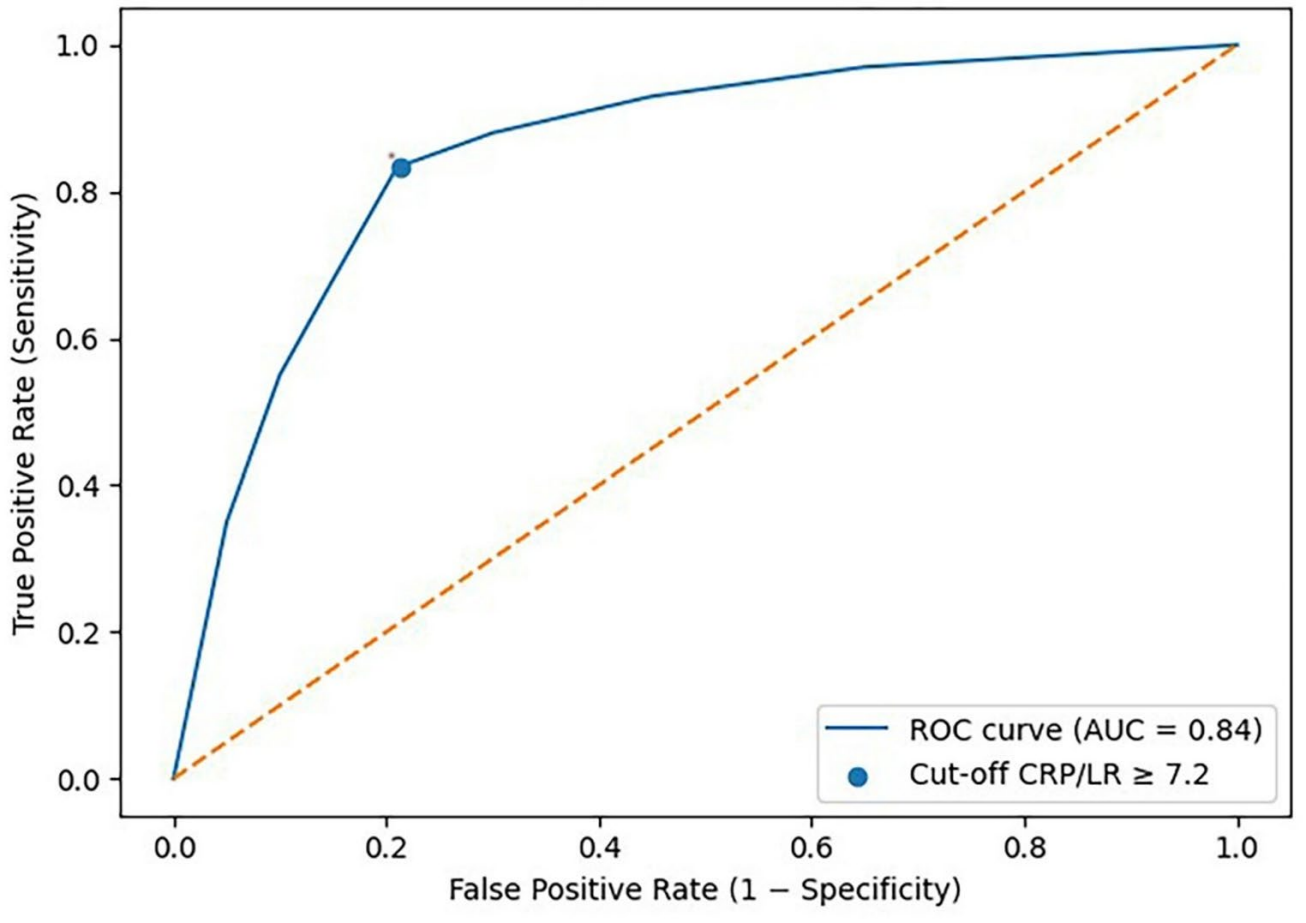
Receiver operating characteristic (ROC) curve illustrating the diagnostic performance of the C-reactive protein–to–lymphocyte ratio (CRP/LR) for predicting appendiceal perforation. The area under the curve (AUC) was 0.84 (95% CI 0.78–0.90), reflecting good discriminatory accuracy. A CRP/LR cut-off value of ≥ 7.2, identified using the Youden index, yielded a sensitivity of 83.3% and a specificity of 78.6%

Preoperative inflammatory markers differed significantly between the groups (Table 2). CRP levels were markedly higher, while lymphocyte percentages were significantly lower in the perforated AA group (both *p* < 0.001). Total leukocyte and neutrophil counts were also elevated in the perforated AA group (*p* < 0.01), and the NLR was significantly higher (*p* < 0.001). Notably, the CRP/LR showed the greatest separation between the groups and demonstrated superior performance over individual inflammatory markers (*p* < 0.001). ROC curve analysis demonstrated good discriminative performance of CRP/LR (AUC 0.84, 95% CI 0.78–0.90). (Fig. 1). Using the Youden index, an optimal CRP/LR cutoff of ≥ 7.2 was identified, yielding a sensitivity of 83.3% and a specificity of 78.6%, highlighting its potential utility for early preoperative risk stratification (Table 3).

**Table 2:** Comparison of preoperative inflammatory markers between the two groups

Parameter	Non-perforated appendicitis (*n* = 290)	Perforated appendicitis (*n* = 48)	*p*-value
CRP (mg/L), median (IQR)	35.2 (18.6-61.4)	78.3 (49.7-121.6)	<0.001
WBC (×10⁹/L), median (IQR)	14.4 (11.9-17.1)	17.0 (14.6-19.8)	0.004
Neutrophils (%), median (IQR)	75.7 (69.2-82.4)	85.6 (79.8-90.2)	<0.001
Lymphocytes (%), median (IQR)	10.5 (7.8-14.3)	6.1 (4.2-8.4)	<0.001
NLR, median (IQR)	6.7 (4.9-9.8)	13.9 (9.6-18.7)	<0.001
CRP/LR, median (IQR)	3.1 (1.8-5.6)	15.1 (9.2-26.4)	<0.001

Continuous variables are expressed as median values (interquartile range) due to non-normal distribution. Between-group comparisons were conducted using the Mann–Whitney U test. CRP/LR was calculated as the ratio of serum C-reactive protein (mg/L) to lymphocyte percentage on admission. *p*-values < 0.05 indicate significance

**Table 3:** Receiver operating characteristic (ROC) curve analysis was used to assess the diagnostic performance of CRP/LR for predicting appendiceal perforation. The optimal cut-off value was calculated using the Youden index. Sensitivity, specificity, positive predictive value (PPV), and negative predictive value (NPV) were calculated with corresponding 95% confidence intervals (CI), where applicable

Metric	Value (95% CI)
Area under curve (AUC)	0.84 (0.78-0.90)
Optimal cut-off (Youden index)	CRP/LR ≥ 7.2
Sensitivity (%)	83.3
Specificity (%)	78.6
Positive predictive value (%)	39.8

After adjusting for age, symptom duration, and body temperature at admission, CRP/LR remained an independent predictor of appendiceal perforation (*p* < 0.01). In contrast, conventional inflammatory markers lost significance, underscoring the superior predictive value of CRP/LR (Table 4).

**Table 4:** Multivariate logistic regression analysis was performed to identify independent predictors of appendiceal perforation. The model was adjusted for age, symptom duration, and body temperature on admission

Variable	OR	95% CI	*p*-value
CRP/LR	1.18	1.10-1.27	<0.001
Age	1.03	0.96-1.11	0.41
Symptom duration ≥24 h	1.61	0.88-2.94	0.12
Body temperature on admission	1.09	0.92-1.28	0.30

Results are presented as odds ratios (ORs) with 95% confidence intervals (CIs). A two-tailed *p*-value < 0.05 was considered significant

## Discussion

The findings of the present study demonstrate that CRP/LR is a robust and independent predictor of appendiceal perforation in children. This composite index showed good discriminative performance and remained significantly associated with perforation after adjustment for relevant clinical variables. By integrating an acute-phase reactant with an immune-response marker, CRP/LR provides a clinically meaningful reflection of disease severity and may improve preoperative risk stratification beyond conventional laboratory parameters. Serum CRP and the WBC count are commonly used to evaluate AA. Elevated CRP is associated with more severe forms of AA, such as perforation, abscess, and phlegmon, in both pediatric and adult patients [9, 16, 17]. However, CRP alone lacks specificity, as it becomes elevated with many inflammatory conditions [18]. Similarly, although leukocytosis and neutrophilia are commonly observed in AA, when considered individually, both demonstrate high specificity but low sensitivity for differentiating simple from perforated AA [9, 19].

Composite indices that integrate multiple aspects of the host inflammatory response, such as the NLR, have been proposed to improve diagnostic accuracy in AA. An elevated NLR correlates with disease severity and perforation in children [11, 12], reflecting a shift toward innate immune activation with relative lymphocyte depletion [20]. A recent pediatric study found that in addition to NLR, other inflammatory indices, such as the platelet-to-lymphocyte ratio (PLR) and the monocyte-to-lymphocyte ratio (MLR), were significantly higher in children with complicated AA than in those with uncomplicated disease, highlighting their value as markers of increasing disease severity [21]. Building on these observations, our findings show that the CRP/LR provides additional discriminatory value. By combining an acute-phase reactant with a measure of lymphocyte suppression, CRP/LR captures complementary information on both inflammatory burden and immune dysregulation. In our cohort, CRP/LR outperformed individual inflammatory markers and other leukocyte-based ratios, showing the greatest separation between non-perforated and perforated AA. These results align with previous studies on pediatric and adult populations, supporting the utility of CRP/LR as a reliable marker of complicated disease [22, 23]. One aspect of our study that deserves attention relates to how the CRP/LR ratio was calculated. We used the lymphocyte percentage, whereas other studies, including that by Alper et al. [23], used absolute lymphocyte counts. This difference seems to explain why reported cut-off values vary among studies. Using the lymphocyte percentage offers practical advantages, as it is part of the standard differential blood count. It provides insight into the balance of immune cell types, which may make the measure more stable despite fluctuations in overall leukocyte numbers. It also highlights a broader challenge in the field; namely, without a standardized method, it is difficult to compare results across studies directly. Harmonizing these approaches in future research would not only make comparisons more meaningful but it would also help translate findings more smoothly into everyday clinical practice. The consistent performance of CRP/LR across age groups and its demonstrated relevance in other infectious, inflammatory, and oncologic conditions support CRP/LR as a robust indicator of systemic inflammation [24–27]. However, a key point to consider is the natural variation in lymphocyte values as children grow. Both absolute counts and relative percentages change considerably throughout childhood, especially in the early years of life. While CRP/LR remained a strong independent predictor even after adjusting for age, applying a single cut-off across all pediatric age groups may not fully capture these developmental differences. As a result, the proposed threshold should be interpreted with care, and using age-specific cut-offs could offer greater diagnostic accuracy. Future prospective studies are warranted to validate these age-adjusted thresholds and to further refine how CRP/LR can be applied in the clinical management of pediatric appendicitis.

The rationale for using CRP/LR lies in the complementary biological information captured by its components. CRP, produced by the liver in response to interleukin-6 and other pro-inflammatory cytokines, serves as a sensitive marker of systemic inflammation [12]. On the other hand, lymphopenia reflects stress-induced suppression of adaptive immunity and has been associated with severe bacterial infections and worse outcomes for inflammatory conditions [13]. Thus, an elevated CRP/LR reflects both heightened systemic inflammation and diminished lymphocytic reserve, a pattern consistent with advanced disease such as perforation. Similar composite indices have proven useful in other settings. For example, the CRP-to-albumin ratio predicts adverse outcomes in sepsis and surgical patients [14], and ratios including lymphocyte counts have prognostic value in colorectal cancer and inflammatory bowel disease [16, 17]. Our findings extend this concept to pediatric AA, suggesting that CRP/LR may serve as a reliable biomarker for perforation.

ROC analysis showed that CRP/LR had a higher area under the curve (AUC) than individual markers, including CRP, lymphocyte proportion, and NLR. The optimal CRP/LR cut-off provided a balanced sensitivity and specificity, highlighting its potential as a clinically useful threshold for preoperative risk stratification. In emergency settings where imaging may be inconclusive or unavailable, incorporating CRP/LR into clinical assessment could help identify children at risk of perforation earlier, guide timely surgical referral, and potentially reduce complications from delayed intervention. Notably, multivariate analysis confirmed that CRP/LR remained independently associated with perforation after adjusting for age, symptom duration, and admission temperature, emphasizing its value beyond conventional clinical predictors. The time-dependent behavior of CRP is an important factor to consider when interpreting CRP/LR values. In our cohort, patients with perforated AA more often had a longer duration of symptoms, which may account in part for the higher CRP/LR observed in this group. Although CRP/LR remained independently associated with perforation after adjustment for symptom duration, its diagnostic performance is likely influenced by the timing of presentation. In the early phase of the disease, particularly within the first 24 h, the CRP levels may not have peaked, which could reduce the marker’s sensitivity. For this reason, CRP/LR should be interpreted alongside symptom duration and the overall clinical context.

Several limitations of this study should be acknowledged. Its retrospective design is prone to selection bias and precludes causal inference. Despite adjustment for key confounders, unmeasured factors such as socioeconomic status, timing of antibiotic administration, and inter-observer variability in operative diagnosis may have influenced the results. Moreover, conducting the study at a single tertiary-care center may limit the generalizability of the findings. Prospective multicenter studies are needed to validate CRP/LR cut-offs and compare their performance with imaging modalities such as ultrasonography and computed tomography. Although intraoperative findings and histopathological confirmation were used to define perforation in this study, these two assessments do not always align. Intraoperative evaluation is, by its nature, influenced by the surgeon’s assessment, albeit one that is not immediately available. This potential lack of concordance, together with inter-observer variability, may have affected case classification and should be considered a limitation of the present study. Age-related variation in lymphocyte values should also be considered when interpreting our findings. Both absolute counts and relative proportions change throughout childhood in normal immune system development. Although CRP/LR remained independently associated with perforation after adjustment for age, using a single cutoff value may not fully capture these physiological differences across all pediatric age groups. In this context, age-specific thresholds may offer greater diagnostic precision and merit further investigation in future studies.

In conclusion, CRP/LR is a readily obtainable and promising biomarker with superior diagnostic performance for distinguishing perforated from non-perforated AA in children. Its incorporation into preoperative assessment may improve risk stratification, guide surgical decision-making, and enhance clinical outcomes. Further prospective research is warranted to confirm these findings and define their potential role in clinical algorithms.

## Data Availability

The datasets presented in the current study are available from the corresponding author on reasonable request.
